# Application of Fractal Image Analysis by Scale-Space Filtering in Experimental Mechanics

**DOI:** 10.3390/jimaging8090230

**Published:** 2022-08-26

**Authors:** Anna Bauer, Wolfram Volk, Christoph Hartmann

**Affiliations:** Chair of Metal Forming and Casting, Technical University of Munich, Walther-Meissner-Strasse 4, 85748 Garching, Germany

**Keywords:** scale-space filtering, fractals, image analysis, experimental mechanics, deformation

## Abstract

Increasingly complex numerical analyses require more and more precise, accurate and varied input parameters in order to achieve results that are as realistic and reliable as possible. Therefore, experimental analyses for material parameter identification are of high importance and a driving force for further developments. In this work, opportunities by applying fractal analysis to optical measurement data of a shear cutting process are investigated. The fractal analysis is based on a modification of the concept of scale-space filtering. Scale exponent fields are calculated for the image sequences of the shear cutting process that are taken by a mobile microscope. A least-square approximation is used for the automated evaluation of the local scale exponent values. In order to determine the change of the scale exponent of individual material points, a digital image correlation is applied.

## 1. Introduction

Numerical procedures, such as simulations or data-driven models, are increasingly utilized for the evaluation and the design of complex systems and components, which can no longer be treated analytically [1,2,3]. In order to obtain meaningful results from the simulations and numerical models, it is important to understand the underlying system and to set the boundary conditions appropriately. In this context, the applied material properties are of high importance [4]. In most cases, the material behavior is determined by experiments, e.g., tensile or bending tests [5]. For the purpose of recording the measurement data, optical measurement systems are used increasingly. The video sequences provided by these optical systems open up numerous new possibilities for evaluation to extract additional information. Most of them, including fractal analysis, have not yet been exhausted. Fractal Analysis in specific is mostly used in static analysis, e.g., surface topology in the context of fracture [6,7,8,9].

### 1.1. The Concept of Fractals

Fractal image analysis is based on the concept of fractals established by Mandelbrot [10]. Fractals are self-similar objects that are too irregular to be described by classical Euclidean geometry. Instead, they have a broken rational dimension—the fractal dimension, being a generalization of the Euclidean concept of dimension [11]. In general, a subdivision into mathematical and natural fractals is made [12]. Mathematical fractals, such as the Koch curve or the Cantor set, are mathematical structures formed by recursive functional rules [13]. The result is an object with details on all scales. In contrast to mathematical fractals, natural fractals describe repeating pattern of real world entities that are not strictly self-similar and hence cannot be defined by recursive functional rules. Their self-similarity exists in a statistical sense and usually extends over a limited range of scales [14,15].

### 1.2. Fractal Dimension

The fractal dimension $D_{f}$ describes the change of the mass $m=\frac{1}{n(l)}$ of the object with scaling *l*. n(l) can be associated with the number of self-similar shapes at scaling *l*. The designation mass refers to all the terms length, area and volume. The fractal dimension of mathematical fractals can be easily calculated by applying the function rule

(1) $$D_{f}=-\frac{\ln(n(l))}{\ln(l)}$$

according to Mandelbrot [10], however this formula does not apply to natural fractals. Due to the statistical self-similarity of natural fractals, approximation approaches, such as differential box-counting [16,17] and probability-based box-counting [18,19], the blanket method [10,20], Brownian motion [18,21] and scale-space filtering [14,22,23,24], are needed for the determination of their fractal dimension.

Since real objects, as well as their images, have only a limited resolution [25], which is either determined by the object itself [15] or limited by the sampling rate of the image, their scaling behavior is finite. However, many of the above-mentioned approaches do not consider the lower scale limit given by the limited resolution, although a consideration of the scale behavior or the fractal dimension below this limit is no longer meaningful. On the contrary, it can even lead to an incorrect calculation of the scale behavior. Scale-space filtering offers the possibility to determine and set the scale limits of a structure. Subsequently, the scale behavior within these limits can be analyzed, and an erroneous evaluation can be prevented [14]. Furthermore, it offers the possibility of analyzing gray-scale images in contrast to simple logical data and thus prevents a significant loss of information through conversion to binary images.

## 2. Scale Space Filtering

In the context of image analysis, the term scale space refers to a family of images representing the original image on different scales [25]. To achieve this representation, the image is gradually convolved with a parameterized filter kernel. After the smoothing operation, the scale behavior is extracted from the resulting signal characteristics with respect to the filter parameters. In order to obtain meaningful results, the convolution cores used must meet certain requirements: the smoothed signal should adopt well-defined values for the limit values of the scale parameter 0 and *∞*. Further, extreme values that occur once must not disappear within finer scales. According to reference [22], the Gaussian bell function is the only function that fulfills these criteria for one-dimensional signals. Reference [23] cites the proof for the uniqueness of the Gaussian function also for two-dimensional signals. For discrete images, however, there is no guarantee that the previously described requirements will be met. In some cases, the discrete convolution operation can also lead to violations of these conditions [25]. This should be kept in mind when applying scale-space filtering to discrete images.

**Figure 1:**
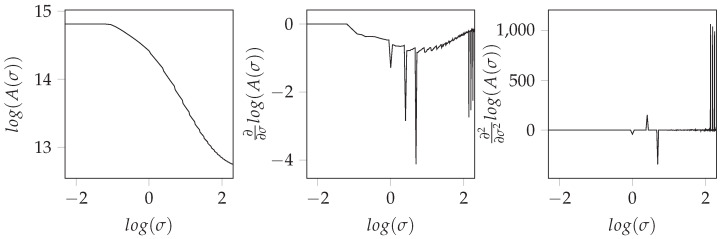
First- and second-order derivatives of the area relation calculated by FDM.

Using the Gaussian filter for the smoothing process, the standard deviation σ, also known as filter width, controls the size of the filter core and thus the filter effect. By varying σ, the image signal *W* is filtered to different degrees $W_{\sigma }$ and can then be analyzed on different scales [26,27]. The scale-space evaluation is based on the gray value intensity surface spanned by the discrete intensity values of the individual pixels for various filter parameters σ [14,24]. To calculate the surface area

(2) $$A\left(\sigma \right)=\int \int \sqrt{1+{\left(\frac{\partial W_{\sigma }}{\partial x}\right)}^{2}+{\left(\frac{\partial W_{\sigma }}{\partial y}\right)}^{2}}dydx,$$

the partial derivatives $\frac{\partial W_{\sigma }}{\partial x}$ and $\frac{\partial W_{\sigma }}{\partial y}$ of the filtered image $W_{\sigma }$ with respect to the horizontal and vertical dimensions of the image plane *x* and *y* are used. Determining the surface content A(σ) for different filter parameters σ according to Formula (2), a relation between the surface and the used filter level can be set up. Plotting the relation of the surface content A(σ) with respect to the corresponding filter parameters σ in a double logarithmic plot, as it is shown in the leftmost plot of Figure 1, there is a region that shows an almost linear behavior for images depicting fractal structures. The slope of this region gives an indicator of the scale behavior of the underlying structure. Furthermore, the relation, also known as the Richardson plot, shows limit values for σ→0 and σ→∞ like it was demanded from the requirements of the convolution core.

### 2.1. Scale Exponent Approximation

According to reference [24], the scale exponent is defined by the slope of the surface relation in the scale range. Hence, for automatic determination of the scale exponent, the derivation of the double-logarithmic surface-relation is necessary. Utilizing of the finite difference method (FDM) for the derivation leads to significantly increased noise. An example of the noise amplification is given by Figure 1, depicting the first and second derivations of a surface-area relation, as it was achieved by Formula (2). A promising alternative is the approximation of an analytic function to the data points of the surface-area relation since the analytical derivations do not suffer from noise amplifications, and meaningful results can be obtained. Here, a sigmoidal function

(3) $$f\left(x\right)=a\cdot \frac{1}{1+e^{-b(x-c)}}+d$$

with the parameters *a*, *b*, *c* and *d* is conducted for the nonlinear least squares approximation. The parameter *a* scales the function. By varying the parameter *b*, the slope and thus the width of the transition region of the sigmoid function can be adjusted. The parameter *c* defines the horizontal shift of the turning point, compared to x=0. The vertical shift is determined by parameter *d*. Compared to using FDM, it is possible to calculate the derivations from the approximation of the parameterized sigmoid function analytically by

(4) $$\frac{\partial f(x)}{\partial x}=ab\cdot \frac{e^{b(x-c)}}{{1+e^{b(x-c)}}^{2}}\;\mathrm{and}$$

(5) $$\frac{\partial^{2}f\left(x\right)}{{\partial x}^{2}}=-ab^{2}\cdot \frac{\left(e^{b(x-c)}-1\right)e^{b(x-c)}}{{\left(1+e^{b(x-c)}\right)}^{3}}.$$

**Figure 2:**
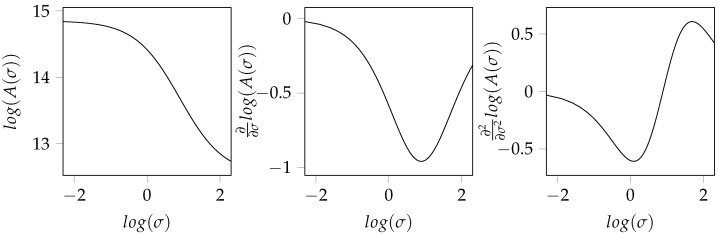
First- and second-order analytically calculated derivatives of the area relation approximated by nonlinear least square approximation of a sigmoid function.

In this way, the amplification of the high-frequency components of the signal is eliminated. The approximation of the surface relation that was numerically derived in Figure 1 is shown in Figure 2 together with its first and second analytically calculated derivatives. In order to obtain repeatable estimates of the scale exponent, the slope at the turning point of the sigmoid approximation is applied, as it can be calculated by Equations (4) and (5).

**Figure 3:**
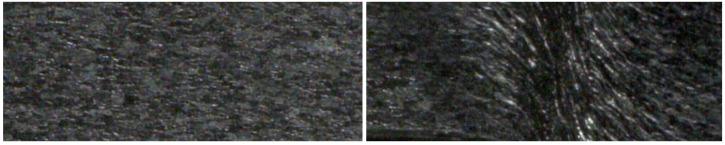
Undeformed image (left) and deformed image during the separation process.

For a meaningful approximation of the area relation, it is important to select suitable filter steps Δσ for scale-space filtering as well as the limits of the filter steps $\sigma_{min}$ and $\sigma_{max}$. The minimum filter level $\sigma_{min}$ is given by the plateau of the sigmoid function in accordance with reference [24]. Since this plateau varies only slightly for different textures, it is set to $\sigma_{min}=0.1$. In order to determine the filter step size Δσ and the upper limit for the analysis of the image sequence at hand, a convergence study regarding two representative images was conducted, one according to an undeformed image, the other regarding a highly deformed image; see Figure 3. According to these results as well as considering the computational effort Δσ=0.1, $\sigma_{max}=10$ were selected for further analyses.

### 2.2. Scale Exponent Field

**Figure 4:**
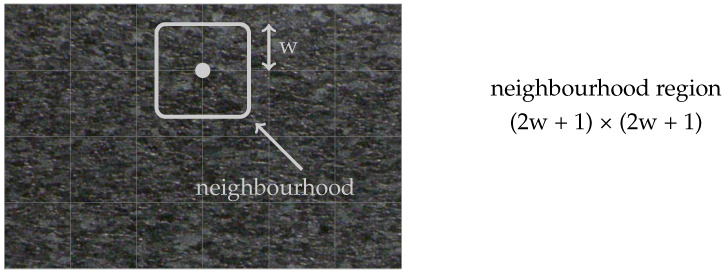
Grid and image excerpt selection for local scale-space analysis.

In principle, an image not only consists of a homogeneous structure, but unites different textures of different scale behavior. Hence, the calculation of one global scale exponent for the entire image is often not suitable for detailed analyses. In order to tackle this issue, scale-space filtering can be adapted to selected parts of the image, allowing for the estimation of local fractal characteristics. Performing the analysis on a grid-like structure as depicted in Figure 4 results in a scale exponent field, giving the spatial fractal characteristics of the image. The quadratic neighborhood region (2w+1)×(2w+1) around the central grid point defines the local evaluation region. The grid size enables an adjustment according to the resolution of the image.

## 3. Scale Exponent and Fractal Dimension

In general, no connection between the scale exponent and the fractal dimension is given [14]. For means of interpretation, a rough estimate of the relation between the scale exponent and fractal dimension is examined using artificial fractal textures with known fractal properties. For the generation of artificial fractal structures, different programs are available [28,29,30]. The program Gwyddion, for example, allows the generation of fractal images by specifying the Hurst exponent and some random seed [30].

**Figure 5:**
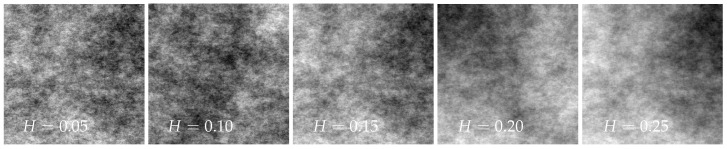
Artificial fractal structures for different Hurst exponents.

**Figure 6:**
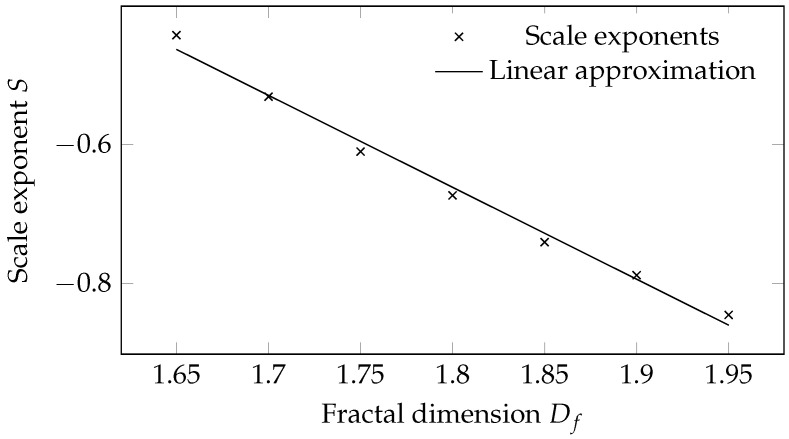
Relation between fractal dimension $D_{f}$ and the scale exponent *S* for artificial fractal structures derived from different Hurst exponents *H*.

Figure 5 shows five exemplary textures generated with Gwyddion using Hurst exponents between H=0.05 and H=0.25. The Hurst exponent *H* is directly related to the fractal dimension $D_{f}=2-H$ [31]. Calculating the scalar exponent via the proposed method in Section 2, the relationship between the scale exponent and the Hurst exponent or the fractal dimension can be obtained. The results are visualized in Figure 6, depicting an approximately linear relationship between the fractal dimension $D_{f}$ and the scale exponent *S*. In this sense, the scale exponent can be seen as a fractal indicator. The proposed evaluation method is implemented in MATLAB.

## 4. Experiments

The approach of scale-space filtering is applied to image sequences of a shear-cutting process of a steel specimen. Shear cutting is an important process in the chipless separation of metallic materials, which commonly shows ductile behavior [32]. In terms of quality assessment, the formation of the cutting surface is important [33]. Many present investigations deal with the influence of different process parameters, i.e., cutting edge geometry, cutting speed and clearance, on the formation of the cutting surface [34]. Material failure occurs when the shear fracture limit is reached, which depends on the deformation and the stress state. Thereby, the material behavior is decisively determined by the strain, strain rate and temperature [35,36]. These variables interact in the shear-cutting-affected zone during the cutting process [37,38]. The aim of the investigations is to track the change in the scale exponent field as a function of the imposed deformation in order to obtain information about the underlying processes.

**Figure 7:**
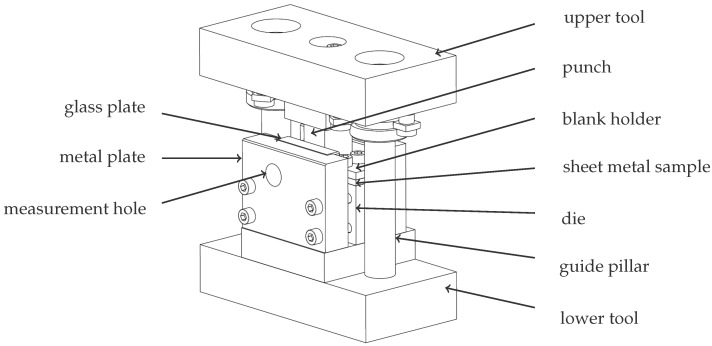
Shear cutting tool for plane strain deformation measurement.

The test setup for the shear cutting experiment is shown in Figure 7. The tool consists of an upper and a lower tool part, which are positioned to each other via guide pillars. Between the upper and lower tool, the sheet metal sample is clamped on a die by means of a blank holder. The punch is mounted above the die in the upper tool. With the vertical movement of the upper tool, the punch lowers onto the specimen and separates the sheet metal. The metal plates serve to fix the specimen to the side. The glass plate placed between the specimen and the metal plate is necessary to ensure the observation of a plane strain state through the measuring hole [39].

**Figure 8:**
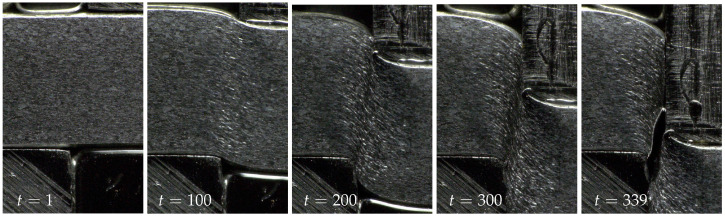
Image sequence of the sheet metal deformation and separation process.

The deformation of the sheet metal edge surface is observed through the measurement hole with a mobile microscope of type Keyence VHX (Osaka, Japan). In order to ensure proper data storage, the experiment was stopped several times. Figure 8 shows some representative images of this experiment. In case of the shear cutting experiment shown in Figure 8, a sample sheet metal of mild steel S355MC with a thickness of 4 mm was used. Further sample preparation was performed by etching the ground edge surface in order to bring out the grain structure of the material. This grain structure not only provides the basis for the subsequent fractal analysis, but also acts as a speckle pattern for calculating the displacements using digital image correlation (DIC) [40,41,42]. The etched sheet metal samples were separated using low punch speeds of 10 mm/min. The time references given at the lower edge of the images in Figure 8 serve the purpose of assigning the deformation to the scale exponent values of the result part. With t=1, the first analyzed image is referred to.

## 5. Experimental Results

**Figure 9:**
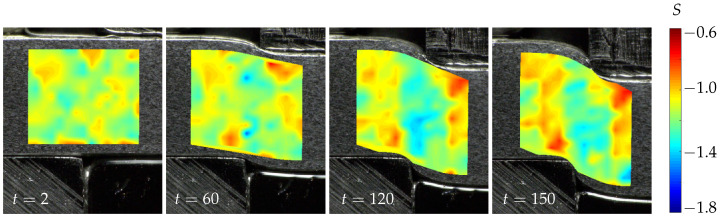
Temporal evaluation of scale exponent fields during shear cutting.

In order to analyze the qualitative progression of the scale exponent field over the course of the shear-cutting process, the fractal analysis was combined with a displacement calculation using DIC, where the abort of the DIC routine leads to the end of the entire evaluation. More information on the DIC approach used can be found in Refs. [41,42]. Figure 9 depicts the scalar exponent fields for the region of interest (ROI) defined in the leftmost image. Due to reflections caused by the deformation of the sheet metal sample, especially in the shear region, the deformation estimation suffers from calculation artifacts. Additionally, the intermittent character of the experiment and the change in illumination on the resumption of the experiment cause those difficulties. However, since the artifacts are limited to the marginal areas, a meaningful assessment of the scale space behavior is still possible. In the case of the unloaded state, i.e., the leftmost image of Figure 9, the scalar exponent values are distributed in a relatively homogeneous manner, exhibiting values around S≈−1.2. In the following time step that is depicted, nests with decreasing scale exponent values of S≈−1.4 are formed at the upper and lower edges of the shear zone. These nests merge to a vertical region that extends along the shear zone in the further progress of the shear-cutting experiment. For laterally adjacent regions, the scale exponent values are increased slightly. Toward later time steps, i.e., the rightmost image depicted in Figure 9, the scale exponent values of the shear region increase slightly. Nevertheless, the shear region is well separated from the undeformed regions by the difference in scale exponent values. From time step *t* = 60 onward, a certain point symmetry of the scale exponent values to the center of the shear zone can be noted. This symmetry may have its origin in the boundary conditions, resulting from the shear-cutting process.

**Figure 10:**
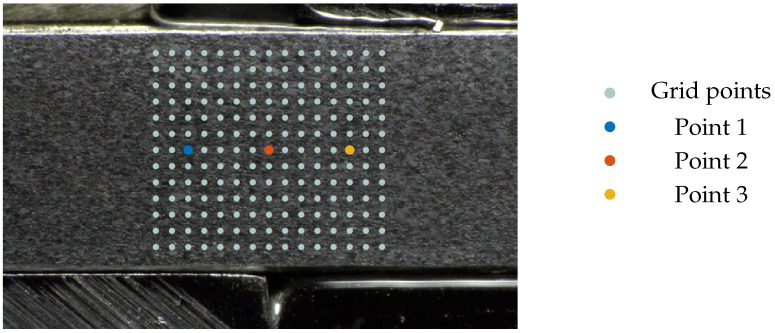
Material points selected or the visualization of the temporal course of the scale exponent.

With these dimensional images of the scale exponent field, only an estimation of the development of the spatial distribution of the scale exponent values but no exact statement can be made about the temporal course of the values of individual material points. Hence, in order to evaluate the fractal behavior over the course of the shear-cutting process, three exemplary points were picked—one located in the shear zone, one in the fixed material zone and one in the material zone that is pressed into the die. The selected points are depicted in Figure 10.

**Figure 11:**
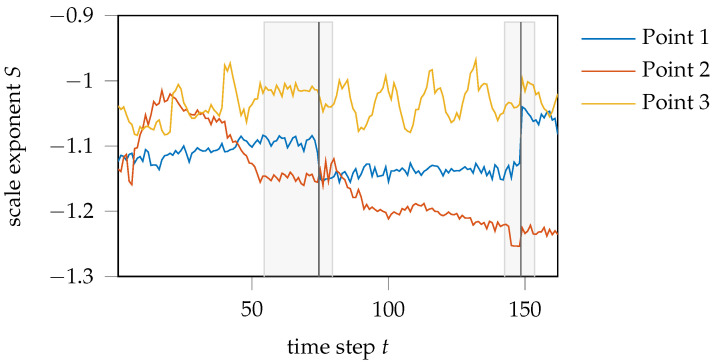
Temporal development of scale exponent values during the shear-cutting experiment.

The temporal courses of the scale exponent values of these points are visualized in Figure 11. Here, it should be noted that the shear-cutting experiment was stopped and restarted several times to ensure data storage. In this context, the camera position is slightly changed, resulting in breaks and constant regions in the scale-exponent progression. The areas concerned are highlighted in gray.

In general, a huge difference between the courses of the three points is recognizable. While the scale exponent curve of the point lying in the fixed material area (point 1) remains almost constant, the temporal course of the scale exponent of the point pressed into the die (point 3) forms a sawtooth-like profile for later time steps. This profile is particularly pronounced from time step t≈75 onward. Similar to the scale exponent progression of point 1, the time progression of point 3 remains constant on average. In contrast, the scale exponent of the point lying in the shear zone (point 2) increases at the beginning up to a time step of t≈20. Afterward, ignoring the gray-shaded regions in which no punch movement, and hence, deformation took place, the scale exponents decrease almost continuously until t≈120. Toward the end of the depicted time series, the values of point 2 also reach an about constant plateau.

For means of physical interpretation, an inverse linear relationship between the scale exponent and fractal dimension is assumed, as it was obtained analyzing artificial fractal structures in Section 3. The material section of the fixed sample area hardly undergoes any deformation due to its fixation. Since no deformation takes place that would lead to a change in the grain boundaries and therefore to a variation in the scale exponent progression, an about constant progression of scale exponent values over the temporal course of the experiment seems reasonable for the material points of this region. The jumps in the scale exponent course are directly located at the time steps where the experiment was stopped. These jumps also occur in the courses of points 2 and 3, but they are less pronounced than for point 1. The scale exponent course of material point 2, located in the shear zone, merges into a continuous decrease after a short increase in scale exponent values in the beginning. According to Figure 6, an increase in the scale exponent value is associated with a decrease in the fractal dimension and thus a decrease in the roughness of the speckle structure. Transferring this knowledge to the deformation of the sheet metal grain, this hints to an initial elongation of the grain boundaries before the scalar exponent starts to decrease with the formation of shear bands, causing an increased roughness of the speckle structure. Since the material points in the material region that are pressed into the die exhibit no major deformation, it is reasonable that the course of their scale exponent values, as represented by point 3, is constant, on average. Nevertheless, in contrast to point 1 of the fixed material area, the profile shows a recurring structure, similar to a saw tooth profile. The formation of these saw-tooth-like characteristics could be due to the stick-slip effect experienced by points on the moving side of the material.

## 6. Summary and Conclusions

The primary goal was to evaluate the opportunities by applying fractal analysis to optical data of experimental mechanics. Therefore, an algorithm was implemented based on the concept of scale space filtering proposed by Müssigmann [24]. A reproducible scale exponent determination was achieved by a least-square approximation of the area relation with a sigmoid function. Adapting this approach to image excerpts, the spatial distribution of scalar exponent values could be investigated. Furthermore, the method of scale space filtering was combined with a DIC calculation. This allowed the observation of the change of the scale exponent values at single image points over the time course of the shear-cutting process.

The modified scale-space filtering method was applied to a shear-cutting experiment. Analyzing the scale exponent fields qualitatively, it could be concluded that the major change of scale exponents takes place in the shear zone, starting with the formation of nets that form into a vertical region extending over the shear zone. Further insights were gained, inspecting the scale exponent evaluation of single material points over the course of the shear-cutting experiment by combining the scale exponent estimation with a deformation calculation via DIC. While the material point in the fixed material area remains almost constant, the material point that is pressed into the die shows a sawtooth-like scale exponent course, remaining constant on average. In contrast to that, the scale exponent course of material point located in the shear zone continuously decreases after a short initial increase, resulting in a large total change of scale exponent values over the shearing process.

From the results, it appears that the scale-space filtering presents as a robust, fast and suitable tool for the fractal analysis of experimental image data depicting the deformation of grain structure of ductile materials.

Currently, the analysis is built on the assumption that the scale exponent is linearly related to the fractal dimension. This has been shown for artificially generated fractal images specified by different Hurst exponents. However, the generality of this assumption remains open.

## 7. Outlook

In future work, numerical analysis validation and verification by the experimental evaluations should be focused on. A promising field, which we think this kind of analysis contributes to, is continuum mechanics approaches for fractal material, as presented, for example, in [43,44].

The assumption on the general validity of the relation between fractal dimension and scale exponent should be further investigated. For example, different classes of images could be tested and evaluated on how the relation of fractal dimension and scale exponent appears and which features show the biggest effect.

Further, future research could include the surface topography of the cut edge surface after the shear cutting process. Here, in particular, the relation of fractal fracture surface characteristics and the development of the scale exponent field during material deformation for materials with a different microstructure seems promising.

## Data Availability

Not applicable.

## Abbreviations

The following abbreviations and symbols are used in this manuscript:

*Abbreviations*	
DIC	digital image correlation
FDM	finite difference method
ROI	region of interest
DFG	German Research Foundation
*Symbols*	
*A*	surface area
a,b,c,d	sigmoid function parameters
$D_{f}$	fractal dimension
*f*	sigmoid function
*H*	Hurst exponent
*l*	scaling
*m*	mass
*n*	number of self-similar shape
σ	standard deviation
*S*	scale exponent
*t*	dimensionless time, time step
*W*	image signal
*w*	neighborhood parameter
x,y	spatial coordinates
